# A Comparative Study of Factors Associated with Depression Between “Otaku” and “Non-Otaku” College Students in Korea

**DOI:** 10.3390/ijerph23040440

**Published:** 2026-03-31

**Authors:** HyungSoon Jang

**Affiliations:** Department of Nursing, Hallym Polytechnic University, Chuncheon 24210, Republic of Korea; janghs@hsc.ac.kr; Tel.: +82-33-240-9475

**Keywords:** depression, otaku, Korean college students

## Abstract

**Highlights:**

**Public health relevance—How does this work relate to a public health issue?**

- The prevalence of depression among individuals in their 20s has significantly increased in recent years, making the mental health of college students a critical public health priority.
- This study explores the relationship between Otaku status—characterized by deep, meaningful immersion in specific interests—and depressive symptoms among Korean college students.

**Public health significance—Why is this work of significance to public health?**

- The Otaku group demonstrated significantly lower levels of depression compared to the Non-otaku group, even after controlling for various socio-demographic and psychological covariates.
- Otaku status explained an additional 2% of the variance in depression, highlighting its role as a potential psychosocial protective resource.

**Public health implications—What are the key implications or messages for practitioners, policymakers and/or researchers in public health?**

- Public health interventions for college students should move beyond traditional task-oriented stress management and encourage engagement in activities that provide emotional stability and personal satisfaction.
- Supporting personally meaningful immersion, such as Otaku status, may serve as an effective integrated strategy for preventing depression and enhancing psychological resilience in the young adult population.

**Abstract:**

Depression is a major mental health concern among college students. However, limited research has examined Otaku status as a psychosocial factor associated with depression. This cross-sectional correlational survey study included 206 Korean college students recruited from online communities and content-related exhibitions. Depression was measured using the Patient Health Questionnaire-9 (PHQ-9), and Otaku status was quantitatively assessed using the Otaku Syndrome Scale. Participants were classified into the Otaku group and the Non-otaku group based on objective cut-off scores. Hierarchical multiple regression analyses were conducted to examine differences in depression after adjusting for self-esteem, college adjustment, academic stress, employment stress, and economic status. The Otaku group reported significantly lower levels of depression than the Non-otaku group (t = 2.05, *p* = 0.041). After adjusting for covariates, Otaku status remained a significant predictor of lower depression (B = −1.63, *p* = 0.004). Self-esteem and college adjustment showed the strongest associations with depression. Otaku status may be associated with lower depression levels among college students and should be considered a meaningful psychosocial factor in mental health promotion strategies.

## 1. Introduction

### 1.1. Research Rationale

Modern society has experienced rapid industrialization and advances in technology, which have improved quality of life but also led to increasing mental health problems. Among these, depression is a common disorder characterized by chronicity and a high risk of recurrence [1]. Recent epidemiological data indicate a significant upward trend in depression rates, particularly among young adults in their twenties, making their mental health a critical public health priority. This vulnerability is especially pronounced among Korean college students, who are in a transitional stage between adolescence and adulthood and face rapid biological, psychological, and social changes [2,3].

The vulnerability to depression is especially pronounced among Korean college students due to a unique and highly pressurized socio-cultural landscape. The transition to college in Korea represents a sudden shift from rote-learning secondary education to an environment requiring intense self-regulation [4,5]. Furthermore, Korea’s rigid social hierarchy and intensifying competition for limited employment heighten career uncertainty, leading to severe psychological stress and emotional instability [4,5,6,7].

Previous studies have identified college adjustment, academic stress, and self-esteem as critical predictors of depression [4,5,6,7]. Meta-analytic research specifically highlights that self-esteem has one of the largest effect sizes regarding depression among Korean university students [8,9]. Beyond these factors, recent studies suggest that engagement in personally meaningful and sustained leisure activities functions as a vital psychological resource, reducing stress and enhancing emotional well-being [10,11,12].

Meanwhile, recently, “Otaku (御宅)” or “Deokhu” culture—characterized by deep immersion in specific interests—has become a prominent social phenomenon among young adults in Korea [10]. The term originally referred to individuals who were excessively immersed in specific cultural contents, often carrying a negative stigma associated with social withdrawal in the past [13,14,15,16,17]. However, its meaning has gradually evolved, and it is increasingly used to describe individuals who demonstrate strong enthusiasm and deep engagement with particular domains, often being reinterpreted as a form of “passionate expertise” [11,12].

While some studies suggest that such immersive activities function as a psychological resource that enhances well-being and reduces negative emotions [12,18], the relationship between these activities and mental health remains controversial. A series of previous studies has consistently demonstrated that excessive engagement in certain content can also be associated with elevated levels of depression and anxiety. Despite this ongoing debate, empirical evidence regarding how this cultural phenomenon specifically relates to the depressive symptoms of Korean college students is still limited [12].

To overcome these limitations, the present study adopted a robust methodological approach by conducting a survey on a single, integrated sample of Korean college students and subsequently classifying respondents into an Otaku group and a Non-otaku group based on objective scores from the Otaku Syndrome Scale [19]. This post hoc classification allows for a more rigorous empirical examination of the relationship between immersive engagement and depression while controlling for major psychosocial covariates, such as self-esteem, college adjustment, and academic and employment stress [19]. This structural approach serves to overcome the limitations of previous descriptive research and provides a more valid basis for evaluating whether these activities function as a significant psychosocial resource for mental health among the broader college student population.

### 1.2. Research Objectives

- To examine the general characteristics of Korean college students, depression-related variables (self-esteem, college adjustment, employment stress, and academic stress), engagement in otaku activities, and levels of depression.
- To analyze differences in levels of depression according to the general characteristics of Korean college students, depression-related variables (self-esteem, college adjustment, employment stress, and academic stress), and engagement in otaku activities.
- To examine differences in levels of depression between the Otaku group and the Non-otaku group after controlling for covariates (general characteristics and depression-related variables) that were found to be significant in univariate analyses.

## 2. Methods

### 2.1. Study Design

This study employed a correlational survey design to examine and compare the associations between general characteristics, self-esteem, college adjustment, employment stress, academic stress, and depression among Korean college students who identify as otaku and those who do not.

### 2.2. Participants and Ethical Considerations

**(1)**  Participants

For data collection, the survey was distributed through hobby-related online communities and at the S Expo, which is held once a month. An online survey was administered in parallel using Google Forms or Naver forms via communities related to “animation, figures, comics, music, celebrities, etc.” The survey included the researcher’s email address and contact information so that respondents could inquire about any questions.

The sample size was determined based on prior studies that provide criteria for estimating an adequate sample size according to effect size and the number of predictors in multiple regression analysis [20,21]. Using G*Power 3.1.9.7, with an effect size of 0.10, a significance level of α = 0.05, power of 0.80, and 10 predictors, the minimum required sample size was calculated as 172. Considering possible attrition, a total of 206 questionnaires were distributed, and responses from all 206 participants were included in the final analysis.

**(2)**  Ethical considerations for participants

Data collection was conducted after obtaining approval from the 2025 Common Committee e-IRB (P01-202508-01-042). Prior to participation, the study purpose and procedures were fully explained through an information sheet, and participants were informed that the collected data would be used only for academic and statistical purposes in accordance with Article 33 of the Statistics Act. During the survey, no personally identifiable information (e.g., names) was collected, and anonymity and confidentiality were specified through the informed consent form.

Participants were informed in advance that they could withdraw from the study at any time during the survey without any disadvantage. The researcher was sufficiently familiar with research ethics requirements stipulated in the Bioethics and Safety Act and applied them throughout the study. Collected data are stored in a secure location accessible only to the researcher and will be retained for three years after the end of the study. After the retention period, electronic files will be permanently deleted and paper documents will be destroyed using a shredder.

### 2.3. Measures

**(1)**  Questionnaire on general characteristics

The participants’ general characteristics were assessed using a self-reported questionnaire developed by the researcher based on a review of prior literature. The questionnaire consisted of items including sex, age, grade level, academic major, living arrangement, and economic status. In particular, economic status was measured through the participants’ subjective judgment of their household income level (Low, Middle, or High), and living arrangement was determined by whether the participant resided with their family (Yes/No).

**(2)**  Otaku syndrome

To identify participants who correspond to the “Otaku” profile, the Otaku Syndrome Scale for college students developed by Kwon [19] was employed. This scale was used as a tool to determine whether an individual exhibits the characteristics of an Otaku, defined as a state of deep immersion involving voluntary knowledge acquisition in a specific domain, investment of time and financial resources, and active participation in related creative activities and communities. The instrument consists of 20 items rated on a 5-point Likert scale, encompassing subdomains of behavioral control (7 items), consumption priority (7 items), and dependency (6 items). Based on the standardized criteria of the scale which established the cut-off score of 79 representing the top 12.1% of individuals who prioritize leisure activities over work, according to the 2016 National Leisure Activity Survey in Korea participants with a total score of 79 or higher were identified as the “Otaku group,” while those below this score were classified as the “Non-otaku group” [19]. The internal consistency reliability (Cronbach’s alpha) was 0.88 at the time of development [19] and 0.96 in the present study.

**(3)**  Self-esteem

Self-esteem was measured using the Korean version of the Rosenberg Self-Esteem Scale originally developed by Rosenberg (1965) [22] and translated/adapted by Lee Hoon-jin and Won (1995) [23]. The scale consists of 10 items, and each item is rated on a 5-point Likert scale from 0 to 4. Higher total scores indicate higher levels of self-esteem. At the time of translation/adaptation, internal consistency reliability was reported as Cronbach’s α = 0.92 [23], and reliability in the present study was also 0.92.

**(4)**  College adjustment

College adjustment was measured using the Student Adaptation to College Questionnaire (SACQ) developed by Baker and Syrik (1984) [24], and the college adjustment scale modified and supplemented by Choi (2018) [25]. This instrument comprises three subdomains: academic adjustment, social adjustment, and personal–emotional adjustment, and responses are rated on a 5-point Likert scale. Higher total scores indicate better adjustment to college life. In Choi (2018) [25], internal consistency reliability (Cronbach’s α) for each subfactor was reported to be 0.87 or higher, and reliability in the present study was 0.79.

**(5)**  Employment stress

Employment stress was measured using an instrument based on Jeong (2001) [26], consisting of items reflecting subjective experiences of employment stress in psychological and internal aspects. The instrument comprises 15 items, including anxiety about unemployment, decreased confidence in employment, and negative emotions in daily life, and is rated on a 5-point Likert scale. Higher total scores indicate higher levels of employment stress. In Jeong (2001) [26], internal consistency reliability was reported as Cronbach’s α = 0.83, and reliability in the present study was 0.70.

**(6)**  Academic stress

Academic stress was measured using a scale modified and supplemented by Han (2023) [27] for academic contexts, based on job stress scales used in prior studies by Shin (2012) [28] and Baek (2016) [29]. This instrument consists of 10 items that include tension, psychological pressure, and psychological and physical responses arising from difficulties due to academic demands experienced by college students. Each item is rated on a 7-point Likert scale. Higher total scores indicate higher levels of academic stress. In Han (2023) [27], internal consistency reliability was reported as Cronbach’s α = 0.91, and reliability in the present study was 0.94

**(7)**  Depression

Depression level was measured using the Patient Health Questionnaire-9 (PHQ-9) developed by Kroenke and Spitzer (2001) [30], and the Korean version translated and supplemented by Park (2010) [31]. This instrument comprises nine items covering decreased interest, depressed mood or hopelessness, sleep problems, fatigue, appetite changes, self-devaluation or guilt, decreased concentration, psychomotor retardation, and suicidal ideation, and is rated on a 4-point Likert scale. Higher total scores indicate greater severity of depressive symptoms. In Park (2010) [31], internal consistency reliability was reported as Cronbach’s α = 0.81, and reliability in the present study was 0.90.

### 2.4. Data Analysis

The collected data were analyzed using IBM SPSS/WIN 25.0 as follows. First, descriptive statistics were conducted to examine the participants’ general characteristics, depression-related variables, Otaku status, and levels of depression.

Next, to examine differences in depression according to general characteristics, depression-related variables, and Otaku status, *t*-tests and analysis of variance (ANOVA) were performed. Pearson correlation analyses were also used to identify associations between continuous variables.

Finally, to analyze differences in depression according to Otaku status after adjusting for general characteristics and depression-related variables as covariates, hierarchical multiple regression analysis with dummy variables was conducted.

## 3. Results

### 3.1. Descriptive Statistics of General Characteristics and Otaku Status Among College Students

The general characteristics of the participants are presented in Table 1. A total of 206 Korean college students participated in the study. Regarding sex distribution, 79.1% were female and 20.9% were male. The mean age of the participants was 21.78 ± 2.77 years. The distribution by Grade level was relatively even, with 12.6% in the 1st year, 31.1% in the 2nd year, 26.7% in the 3rd year, and 29.6% in the 4st year. With respect to academic major, 46.1% were in natural sciences, 45.1% in humanities and social sciences, and 8.7% in arts and physical education. Regarding living arrangements, 60.2% of the participants lived with their family, whereas 39.8% did not. Subjective economic status was most frequently reported as middle (72.3%), followed by low (15.0%) and high (12.6%).

Regarding Otaku status, 52.2% of participants were classified into the Non-otaku group, while 47.8% were identified as the Otaku group. Descriptive statistics for the major variables indicated that the mean score for self-esteem was 25.44 ± 8.96, and the mean score for college adjustment was 39.75 ± 8.04. Among stress-related variables, the mean score for employment stress was 46.33 ± 8.46, and the mean score for academic stress was 40.30 ± 15.02. The mean depression score was 17.71 ± 6.19.

### 3.2. Differences and Associations in Depression According to General Characteristics and Otaku Status

Differences in levels of depression according to general characteristics, depression-related variables, and Otaku status are presented in Table 2.

The results showed that there were no significant differences in depression levels according to sex (t = 1.75, *p* = 0.081), age (r = 0.05, *p* = 0.403), grade level (F = 0.58, *p* = 0.632), major (F = 0.67, *p* = 0.515), or living with family (t = 0.41, *p* = 0.683).

In contrast, significant differences or significant associations with depression were found for economic status (F = 6.04, *p* = 0.003), self-esteem (r = −0.72, *p* < 0.001), college adjustment (r = −0.58, *p* < 0.001), employment stress (r = 0.45, *p* < 0.001), academic stress (r = 0.59, *p* < 0.001), and Otaku statu (t = 2.05, *p* = 0.041).

Specifically, depression scores differed significantly according to economic status (F = 6.04, *p* = 0.003). Post hoc analysis indicated that the group with low economic status showed significantly higher levels of depression than the middle and high economic status groups (a > b,c). This result suggests that lower subjective economic status is associated with higher levels of depression.

Correlation analyses among continuous variables showed that self-esteem (r = −0.72, *p* < 0.001) and college adjustment (r = −0.58, *p* < 0.001) were significantly negatively correlated with depression, indicating that higher levels of self-esteem and college adjustment were associated with lower levels of depression. In contrast, employment stress (r = 0.45, *p* < 0.001) and academic stress (r = 0.59, *p* < 0.001) were significantly positively correlated with depression, indicating that higher stress levels were associated with increased depression.

When depression levels were compared according to engagement in otaku activities, the mean depression score of the Non-otaku group was 18.55 ± 5.90, whereas that of the Otaku group was 16.79 ± 6.40. The difference between the two groups was statistically significant (t = 2.05, *p* = 0.041). That is, the Otaku group exhibited relatively lower levels of depression than the Non-otaku group.

### 3.3. Factors Associated with Depression Among College Students: Hierarchical Regression Analysis Including Otaku Status

To examine the association of Otaku status with depression after adjusting for covariates, a hierarchical multiple regression analysis was conducted, and the results are presented in Table 3. Based on the results of the univariate analyses, general characteristics and depression-related variables that showed statistical significance were selected as covariates, and two regression models were constructed. Model I included only the covariates, whereas Model II included Otaku status in addition to the variables entered in Model I. In both models, the variance inflation factor (VIF) values were confirmed to be less than 10, indicating no issue with multicollinearity.

First, Model I was statistically significant (F = 45.74, *p* < 0.001), with an adjusted coefficient of determination (adjusted R^2^) of 0.56. Among the covariates, self-esteem and college adjustment were identified as significant predictors of depression. Specifically, for every one-point increase in self-esteem, the depression score decreased by an average of 0.37 points (B = −0.37, *p* < 0.001), and for every one-point increase in college adjustment, the depression score decreased by 0.12 points (B = −0.12, *p* = 0.024). In contrast, economic status, employment stress, and academic stress did not show significant associations with depression within this model.

Next, in Model II, Otaku status was added to the analysis. The regression model remained statistically significant (F = 41.82, *p* < 0.001), and the adjusted R^2^ increased to 0.58. This indicates that Otaku status explained an additional 2% of the variance in depression after controlling for covariates. In Model II, self-esteem and college adjustment continued to be significant predictors. Specifically, a one-point increase in self-esteem and college adjustment was associated with a decrease in depression scores by 0.37 points (B = −0.37, *p* < 0.001) and 0.12 points (B = −0.12, *p* = 0.025), respectively.

Most importantly, Otaku status was significantly associated with depression. After adjusting for covariates, the Otaku group showed depression scores that were, on average, 1.63 points lower than those of the Non-otaku group (B = −1.63, *p* = 0.004). When comparing the relative importance of variables based on standardized regression coefficients (β), self-esteem (β = −0.54) had the strongest association with depression, followed by college adjustment (β = −0.16) and Otaku status (β = −0.13).

## 4. Discussion

The present study examined the differences in depression levels according to Otaku status among Korean college students, adjusting for significant covariates identified in the previous literature.

First, the proportion of the Otaku group in this study (47.8%) was notably higher than the 30.1% reported in earlier research using the same instrument [18]. This trend likely reflects the recent “digging” culture—a consumer phenomenon of deep immersion in preferred content—which has fostered social acceptance and normalization of Otaku identity among youth [32]. However, the potential for overestimation due to recruitment from content-related exhibitions and online communities should be considered when generalizing these results.

Second, the participants’ mean depression score was 17.71 ± 6.19. While direct comparisons with prior studies [33] are complex due to varying contexts, this score highlights the specific psychological strain currently experienced by Korean college students.

Third, a significant finding was that the Otaku group (16.79 ± 6.40) exhibited lower depression levels than the Non-otaku group (18.55 ± 5.90; *p* = 0.004), even after controlling for major covariates. This aligns with research indicating that individuals who identify as Otaku report fewer negative emotions [12]. Qualitative evidence further supports the notion that Otaku status functions as an “emotional outlet,” facilitating recovery from stress and reducing emotional isolation through meaningful immersion [34,35].

However, the psychological impact of such immersion remains a subject of academic debate. While this study emphasizes adaptive coping, some scholars have argued that excessive immersion can lead to social withdrawal or increased anxiety [13,14,15,16,17]. These conflicting findings suggest that the benefits of being an Otaku may depend on the balance between one’s immersive interests and social functioning [11,12]. In the highly competitive Korean context, Otaku engagement appears to provide a critical psychological buffer, offering stability and satisfaction through specialized interests.

Fourth, in addition to Otaku status, self-esteem and college adjustment were identified as significant predictors of depression. These findings are consistent with prior research involving various student populations [4,6,36,37], which consistently reports that higher self-esteem and better adjustment are robust protective factors against depression.

Interestingly, employment stress and academic stress were not significantly associated with depression in this model. This aligns with previous findings, such as a study of 311 nursing students [38], which indicated that while academic issues are prominent stressors, they often lose predictive power when emotional and interpersonal factors are adjusted.

One explanation is that while academic and employment stress are task-oriented and manageable, depression is more fundamentally rooted in psychological factors like low self-esteem and poor adjustment. Thus, internal resources play a more decisive role in explaining depression than external task-based burdens. Furthermore, the independent effects of these stressors may have been attenuated due to shared variance with other psychosocial covariates, including Otaku status adjusted in the model [38].

Taken together, these findings suggest that understanding depression among Korean college students—who often navigate a highly competitive academic and employment landscape—requires shifting attention from mere task-oriented burdens to internal psychological resources, college adjustment, and the presence of personally meaningful immersion, such as Otaku status. Notably, the lower depression levels in the Otaku group suggest that Otaku status may function as a protective factor in regulating emotional states within this high-stress environment. This implies that depression prevention strategies in South Korea should move beyond task-related stress management toward an integrated approach that enhances self-esteem and college adjustment while actively supporting engagement in meaningful, immersive activities.

The present study has several limitations. First, because this study was designed as a cross-sectional correlational survey, there are inherent limitations in interpreting the relationship between Otaku status and depression in causal terms. Therefore, future research should adopt longitudinal research designs to more clearly examine the causal mechanisms. Second, because participants were recruited primarily from content-related exhibitions and online communities, the proportion of the Otaku group may have been relatively high, and caution is required when generalizing the findings.

Despite these limitations, the present study has significant academic and practical strengths. First, while previous research on Otaku culture has largely been limited to theoretical and socio-cultural analyses or descriptive investigations of engagement patterns, the present study extends the literature by empirically examining the psychosocial impact of Otaku status on mental health, specifically depression, within the unique sociocultural context of Korean youth.

Second, rather than relying on subjective self-identification or qualitative descriptions, this study utilized a standardized measurement tool—the Otaku Syndrome Scale—to quantitatively evaluate and classify Otaku status. By employing objective score criteria to examine its relationship with depression, this study provides robust empirical evidence. Therefore, this study contributes to the field by establishing Otaku status not merely as a cultural phenomenon but as a measurable and meaningful psychosocial factor that plays a significant role in the emotional regulation of Korean college students.

## 5. Conclusions

This study examined the association between Otaku status and depression among Korean college students while controlling for major psychosocial variables. The results showed that Otaku status was significantly associated with lower levels of depression, even after adjusting for self-esteem, college adjustment, academic stress, and employment stress. These findings suggest that within a highly competitive academic and employment environment, engagement in personally meaningful immersion, such as Otaku status, represents a vital psychosocial factor that supports emotional well-being. However, given the cross-sectional design and specific sampling context of this study, further longitudinal research is required to clarify the causal direction of this relationship.

## Figures and Tables

**Table 1:** General characteristics of participants.

Variables	Categories	*n* (%) or M ± SD
Sex	Female	163 (79.1)
	Male	43 (20.9)
Age		21.78 ± 2.77
Grade level	1st	26 (12.6)
	2nd	64 (31.1)
	3rd	55 (26.7)
	4th	61 (29.6)
Major	Humanities and Social Sciences	93 (45.1)
	Natural Sciences	95 (46.1)
	Arts and Physical Education	18 (8.7)
Living with family	Yes	124 (60.2)
	No	82 (39.8)
Economic status	Low	31 (15.0)
	Middle	149 (72.3)
	High	26 (12.6)
Self-esteem		25.44 ± 8.96
College adjustment		39.75 ± 8.04
Employment stress		46.33 ± 8.46
Academic stress		40.30 ± 15.02
Depression		17.71 ± 6.19
Otaku status	Non-otaku group	108 (52.2)
	Otaku group	98 (47.8)

**Table 2:** Differences and Associations in Depression According to General Characteristics and Otaku Status.

Variables	Categories	M ± SD	t or F or r	*p*
Sex	Female	17.69 ± 6.31	1.75	0.081
	Male	15.77 ± 6.71		
Age			0.05	0.403
Grade level	1st ^a^	18.54 ± 5.87	0.58	0.632
	2nd ^b^	17.31 ± 7.05		
	3rd ^c^	18.35 ± 6.37		
	4th ^d^	17.19 ± 5.21		
Major	Humanities and Social Sciences ^a^	17.84 ± 6.42	0.67	0.515
	Natural Sciences ^b^	17.31 ± 5.99		
	Arts and Physical Education ^c^	19.05 ± 6.21		
Living with family	Yes	17.85 ± 6.13	0.41	0.683
	No	17.49 ± 6.32		
Economic status	Low ^a^	21.06 ± 6.98	6.04	0.003 (a > b,c)
	Middle ^b^	17.29 ± 5.84		
	High ^c^	16.12 ± 6.10		
Self-esteem			−0.72	<0.001
College adjustment			−0.58	<0.001
Employment stress			0.45	<0.001
Academic stress			0.59	<0.001
Otaku status	Non-otaku group	18.55 ± 5.90	2.05	0.041
	Otaku group	16.79 ± 6.40		

Grade level: ^a^ = 1st year, ^b^ = 2nd year, ^c^ = 3rd year, ^d^ = 4th year; Major: ^a^ = Humanities and Social Sciences, ^b^ = Natural Sciences, ^c^ = Arts and Physical Education; Economic status: ^a^ = Low, ^b^ = Middle, ^c^ = High.

**Table 3:** Factors Associated with Depression Among College Students: Hierarchical Regression Analysis In-cluding Otaku Status.

Variables	Category	Model I						Model II					
		B	SE	β	t	*p*	VIF	B	SE	β	t	*p*	VIF
Economic status	Low ^a^	Ref.						Ref.					
	Middle ^b^	−0.90	0.83	−0.07	−1.08	0.280	1.69	−0.80	0.81	−0.06	−0.99	0.324	1.70
	High ^c^	0.22	1.15	0.01	0.19	0.848	1.80	−0.05	1.13	0.00	−0.04	0.966	1.81
Self-esteem		−0.37	0.04	−0.53	−8.53	<0.001	1.82	−0.37	0.04	−0.54	−8.83	<0.001	1.83
College adjustment		−0.12	0.05	−0.16	−2.28	0.024	2.34	−0.12	0.05	−0.16	−2.25	0.025	2.34
Employment stress		0.02	0.05	0.02	0.37	0.712	1.93	0.01	0.05	0.02	0.26	0.794	1.93
Academic stress		0.06	0.03	0.14	1.80	0.074	3.00	0.06	0.03	0.14	1.76	0.080	3.01
Otaku status	Non-otaku group							Ref.					
	Otaku group							−1.63	0.57	−0.13	−2.88	0.004	1.03

Economic status: ^a^ = Low, ^b^ = Middle, ^c^ = High. R^2^ = 0.59, Adj. R^2^ = 0.58, F = 41.82, *p* < 0.001.

## Data Availability

The data presented in this study are available on reasonable request from the corresponding author. The data are not publicly available due to privacy and ethical restrictions.
